# Portal Flow Modulation During Liver Transplantation for Acute Liver Failure: A Case Report

**DOI:** 10.7759/cureus.82553

**Published:** 2025-04-18

**Authors:** Pilar Leal-Leyte, Armando Baeza-Zapata, Frida M Mendoza-Jaimes, Jose A Avila-Armendariz, Arturo Luevano-Gonzalez, Daniel Zamora-Valdés

**Affiliations:** 1 Organ Transplantation, Naval Medical Center, Mexico City, MEX; 2 Medicine, Hospital Angeles Chihuahua, Chihuahua, MEX; 3 General Practice, Hospital Angeles Chihuahua, Chihuahua, MEX; 4 Radiology, Hospital Angeles Chihuahua, Chihuahua, MEX; 5 Pathology, Hospital Angeles Chihuahua, Chihuahua, MEX; 6 Hepatobiliary Sciences and Liver Transplantation, King Abdulaziz Medical City, Riyadh, SAU

**Keywords:** acute liver failure, case report, inflow modulation, liver transplantation, portal pressure

## Abstract

Small-for-size syndrome during living donor liver transplantation has been widely studied. Whole allograft deceased donor liver transplantation from small pediatric donors is challenging and may face the same risks and complications. Here, we report a case of an adult patient with acute liver failure who underwent liver transplantation using a pediatric donor graft, combined with splenectomy as portal inflow modulation.

## Introduction

Acute liver failure is a rare, life-threatening clinical syndrome, potentially reversible, characterized by multiple organ failure secondary to the rapid loss of liver function in a patient without preexisting liver disease. It is defined by the presence of jaundice, coagulopathy (international normalized ratio (INR) ≥ 1.5), and hepatic encephalopathy of any degree with a duration of less than 26 weeks [1].

The etiology of acute liver failure should be investigated and treated as appropriate [2]. Acute hepatitis A infection has a favorable prognosis in most cases, and <1% of adult patients progress to acute liver failure [3]. However, among such patients, transplant-free survival is only 70% [2].

Liver transplantation is the standard of care for selected patients at high risk of mortality due to acute liver failure. The availability of a whole allograft from a deceased donor for patients with acute liver failure listed for urgent liver transplantation is an issue in countries with a low donation rate [4]. This has led to innovative strategies to increase the pool of available allografts [5].

Small liver allografts in the setting of liver transplantation have been associated with early allograft dysfunction, small-for-size syndrome (SFSS), and potentially worse patient and graft survival [6]. Intra-operative monitoring of portal hemodynamics and inflow modulation is widely recommended [7]; however, reports on graft inflow modulation in liver transplantation using whole allografts in noncirrhotic patients are limited, particularly in the setting of acute liver failure.

## Case presentation

**Figure 1:**
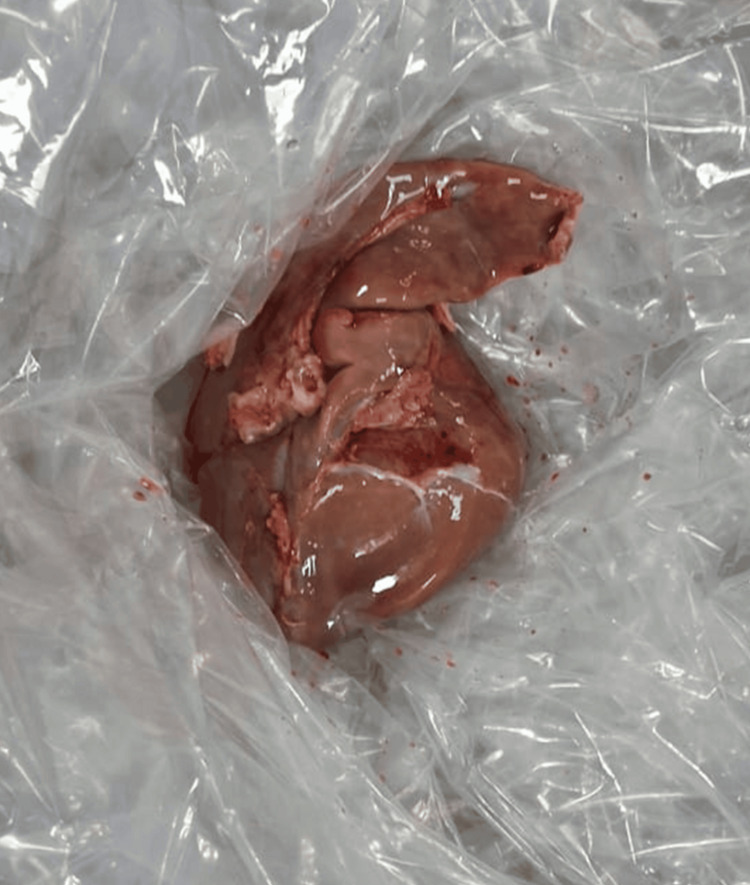
Liver allograft prior to the backtable procedure (weight: 470 g).

The liver of a five-year-old brain-dead donor (Liver Donor Profile Index 1.48) was allocated through a national emergency status offer to a 29-year-old female patient with acute liver failure due to hepatitis A viral infection. The patient had a history of stable chronic kidney disease due to primary focal and segmental glomerulosclerosis. She received medical management for 20 days, including continuous renal replacement therapy and molecular adsorbent recirculating system, however, her neurological status deteriorated. As part of her workup, she underwent transjugular hepatic vein gradient pressure measurement, which was found to be 10 mmHg. The recipient's weight was 59 kg, her standard liver volume was 1,179 cc, and imaging revealed an anatomical variant with the right hepatic artery arising from the superior mesenteric artery. The donor weight was 15 kg, the liver allograft was 470 g, and the anatomy was normal (Figure 1).

Native liver was noncirrhotic, and there was no evidence of significant portal hypertension. Caval-sparing total hepatectomy allowed for side-to-side cavo-cavostomy, followed by standard portal vein reconstruction. The cold ischemia time was 10 hours, and the warm ischemia time was 41 minutes. Reperfusion was normal. The variant right hepatic artery of the recipient was anastomosed with the proper hepatic artery of the donor. The graft-to-recipient weight ratio (GRWR) was 0.79%, and the standard liver volume ratio was 39.8%. Due to the arterial size mismatch and absence of diastolic flow on the hepatic artery during intraoperative Doppler, portal venous pressure (PVP) was measured with a 7 Fr catheter through the superior pancreaticoduodenal vein and was found to be 20 mmHg with a central venous pressure of 10 mmHg (flowmetry is not routinely available in Mexico). Due to the normal size of the splenic artery and spleen, ligation was felt to be insufficient, and splenectomy was performed; immediately, PVP was reduced to 10 mmHg with a central venous pressure of 6 mmHg, Doppler artery examination became normal, bile was seen coming from the donor bile duct, and duct-to-duct anastomosis was performed.

**Figure 2:**
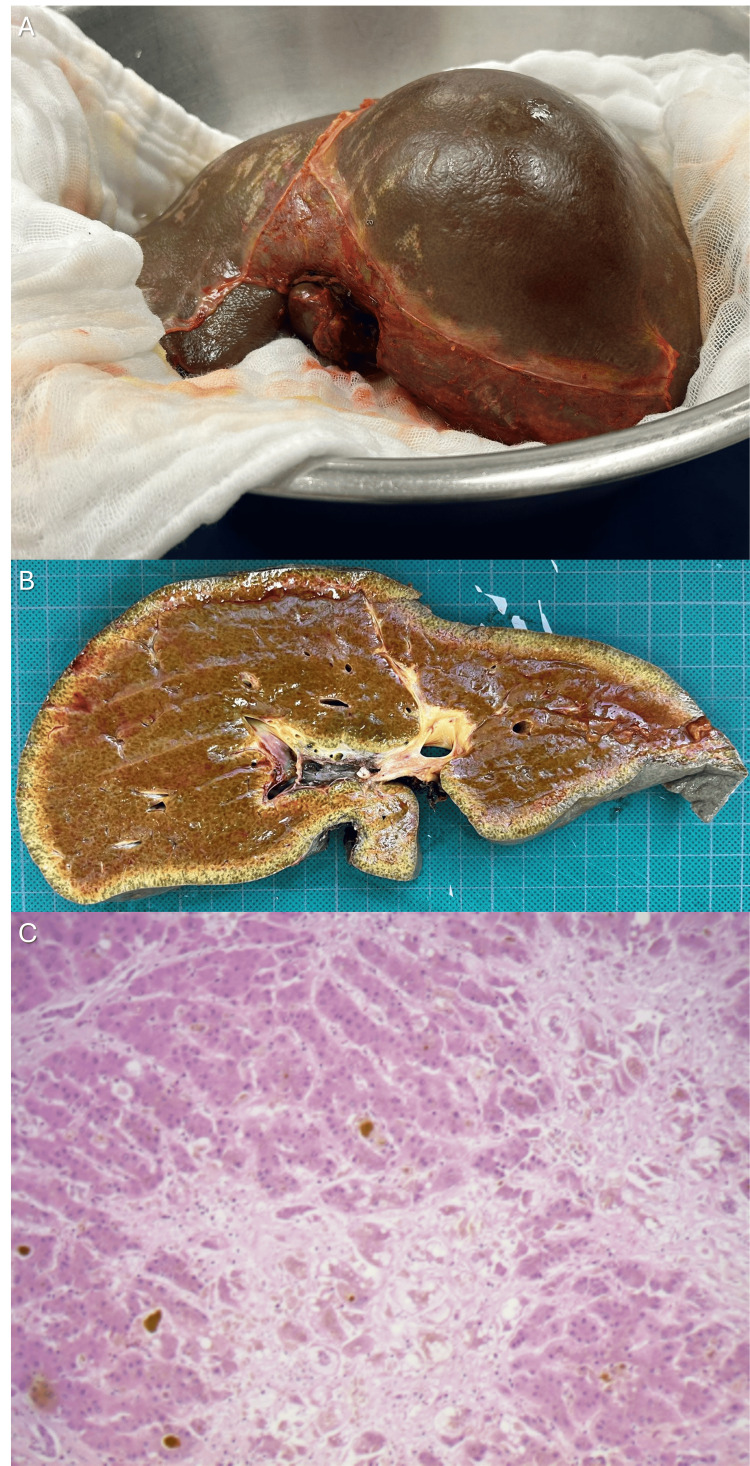
Explanted liver. (A) Operative specimen immediately after resection. (B) Coronal slice of formaldehyde-fixed tissue. (C) Microphotograph (40×) of hematoxylin and eosin-stained slide showing extensive hepatocyte necrosis.

The explanted liver showed massive parenchymal necrosis (approximately 80% of the parenchyma) (Figure 2).

**Figure 3:**
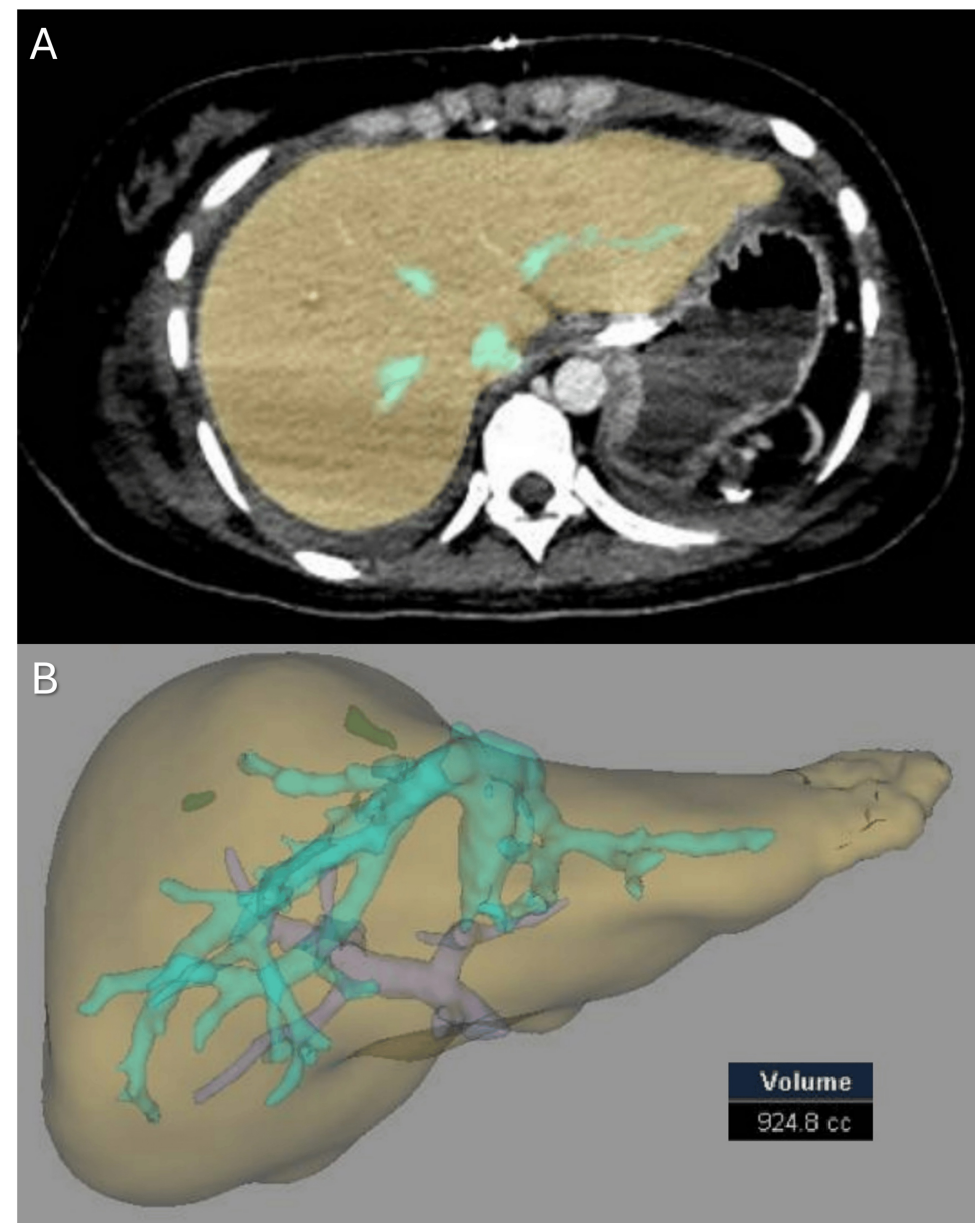
Unenhanced abdominal computed tomography on postoperative day 6 showing (A) no intra-abdominal complications, the presence of an incisional hematoma, and (B) a liver graft volume of 924.8 cc.

The liver graft function was satisfactory, and the patient had an uneventful early postoperative course, leaving the ICU after four days. Six days after transplantation, she developed a painful incisional hematoma for which she underwent an abdominal CT, which showed no intra-abdominal complications and a liver graft volume of 924.8 cc (Figure 3; growth 97%).

**Figure 4:**
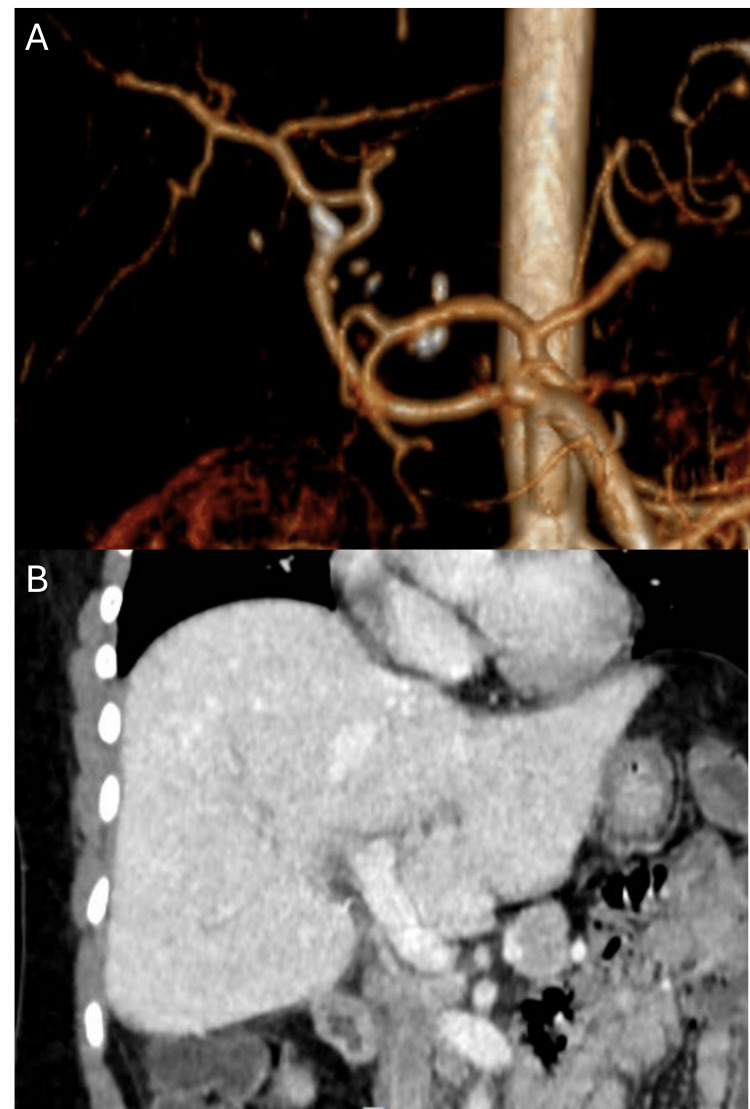
Postoperative abdominal computed tomography with intravenous contrast one year after transplantation. (A) Digital reconstruction of the arterial phase showing a variant right hepatic artery and a patent anastomosis of the graft’s proper hepatic artery. (B) Coronal reconstruction of the portal phase showing a normal liver graft with a patent portal vein.

The follow-up Doppler scans were normal. One month after transplantation, the patient received the appropriate vaccinations needed after splenectomy. At one year follow-up, liver function tests were within normal limits, and abdominal CT showed no abnormalities (Figure 4).

## Discussion

Graft-recipient size mismatch is a major factor associated with graft dysfunction after liver transplantation [8]. This mismatch can be exacerbated by portal hypertension, leading to portal hyperflow, resulting in an arterial buffer response (vasospasm), manifested by decreased systolic arterial flow and absent diastolic flow in the hepatic artery, which may raise concerns about thrombosis [9,10]. Most studies on SFSS focus on living donor liver transplantation, as this scenario is far less common in deceased donor liver transplantation, particularly with whole allografts.

Ideally, liver allografts from deceased pediatric donors should be allocated to pediatric patients. In some circumstances, such as those described in this case report (national priority allocation due to acute liver failure), an adult patient received a healthy but small liver graft. Early reports found that adult recipients of pediatric donors had a higher risk of vascular complications and graft loss, associated with longer ischemia times [11,12]. However, more recent studies have shown that the outcomes of adult recipients of pediatric liver grafts are excellent [10].

Dai et al. reported the case of a 61-year-old cirrhotic patient in Hong Kong who received a liver allograft from a two-year-old donor. The allograft was 360 g, and the recipient weighed 64.5 kg (GRWR 0.56%). The PVP and flow were high, and no inflow modulation was performed. The patient developed SFSS; his INR returned to normal after 11 days, his bilirubin level remained elevated for two months, and he had persistent ascites for 50 days. At the time of the report, the patient was alive with a functional graft, 16 months after the liver transplantation [13]. Bowen et al. reported a 51-year-old, 72-kg, cirrhotic patient in Zhengzhou who received a 420 g whole liver from a pediatric donor (GRWR 0.58%). PVP and flow were not reported; splenic artery ligation was performed after reperfusion, terlipressin infusion was administered for seven days, and the recipient recovered uneventfully [14]. Feng et al. reported a 60-year-old cirrhotic patient in Taiwan who underwent liver transplantation from a pediatric donor. The whole allograft weighed 450 g, and the recipient weighed 60 kg (GRWR 0.75%). PVP and flow normalized after splenectomy, and she had an uneventful recovery [15].

Zhang et al. reported a series of 19 adult patients with cirrhosis in Beijing who received liver allografts from pediatric donors after circulatory death. Portal flow above 250 mL/min/100 g of liver tissue led to splenic artery ligation, and splenectomy was performed when no significant improvement occurred after ligation. One patient underwent splenic artery ligation and five underwent splenectomy. Two patients had small for size/flow syndrome; one had a GRWR of 0.86% who did not undergo inflow modulation, and a recipient of a graft with 70-80% steatosis [16].

Our case illustrates these important concepts. First, modern liver transplant teams must be prepared for major adaptations in urgent situations under donor scarcity. Second, portal hypertension after liver transplantation can occur in patients without chronic liver disease. Patients with acute liver failure may develop portal hypertension related to the degree of liver architectural distortion and their hyperkinetic circulatory state [17]. In our case, the mild portal hypertension of the patient (preoperative gradient 10 mmHg) was exacerbated by the reduced intrahepatic portal vascular bed of the small allograft. Third, PVP and/or flow should be measured when larger recipients receive livers from smaller donors. Fourth, normalization of portal pressure and/or flow through inflow modulation may reduce the risk of SFSS, even when the GRWR is <0.8% [18]. Fifth, side-to-side cavo-cavostomy allows caudal shifting of small allografts, which facilitates vascular and biliary reconstructions.

## Conclusions

We describe a successful case of an adult patient with acute liver failure who received an allograft from a small pediatric donor. Lowering the GRWR limit can definitely expand donor availability, particularly in critical, urgent circumstances. Acute hepatitis A virus infection continues to be an important cause of acute liver failure despite vaccine availability and should be investigated promptly. Patients diagnosed with acute liver failure due to acute hepatitis A should be closely monitored and urgently referred to transplant centers for management.

Intraoperative Doppler evaluation and monitoring of portal hemodynamics allow proper surgical management through the identification of abnormal hepatic artery diastolic flow, reduced overall arterial flow, and portal hyperflow. SFSS can be prevented through timely portal inflow modulation aimed at normalizing portal hemodynamics.
